# Energy efficient UAV-assisted two layer hierarchical bit-mapping access based MAC protocol

**DOI:** 10.1038/s41598-025-31516-x

**Published:** 2025-12-10

**Authors:** Dattaprasad Narayan Golatkar, Manoj Tolani, Smitha N. Pai, Yogesh Vilas Mahadik, Pankaj Kumar, Gaurav Suman

**Affiliations:** 1 Manipal Institute of Technology, Manipal Academy of Higher Education, Manipal, 576104 Karnataka India; 2 Department of Electronics and Communication Engineering, Jaypee Institute of Information Technology, Noida, UP 201309 India; 3 Department of Electrical Engineering, Government Polytechnic, Malvan, Maharashtra India

**Keywords:** UAV-assisted WSN, Energy efficient MAC, Bit-mapping access, Two layer hierarchical clustering, Mission-critical communication, Medium access control, Bandwidth optimization, Engineering, Mathematics and computing

## Abstract

Reliable and efficient medium access control (MAC) protocols are crucial for energy-efficient data communication and effective resource utilization in Wireless Sensor Networks (WSNs). In this work, we propose an Energy-Efficient UAV-assisted Two-Layer Hierarchical (EE-UAV-TLH) MAC protocol, designed for mission-critical applications with energy constraints and limited bandwidth. The protocol employs a two-level hierarchical clustering structure, where sensor nodes communicate with their Cluster Head (CH), and the CH coordinates with an Unmanned Aerial Vehicle (UAV) for data collection and transmission scheduling. By adopting a bit-mapping access scheme, EE-UAV-TLH significantly reduces idle listening and control overheads, while the UAV acts as a mobile sink and synchronization controller, enhancing scalability and minimizing energy usage. Simulation results across diverse scenarios demonstrate that the TLH-ETDMA variant consistently outperforms its counterparts (TLH-TDMA and TLH-BMA), yielding average energy savings of about 20% over TLH-TDMA and 34% over TLH-BMA. In peak cases, EE-UAV-TLH achieves up to 31% improvement against TLH-TDMA and up to 52% against TLH-BMA. Even under large-scale deployment with 110 nodes, it sustains 17% and 39% savings, respectively, highlighting its superior efficiency and scalability for UAV-assisted WSNs.

## Introduction

Wireless Sensor Networks (WSNs) have been an integral part of our daily lives, and they have applications in environmental monitoring, smart agriculture, disaster management, and industrial automation. These networks are made up of sensor nodes that are physically dispersed and that observe and track the conditions in the physical or natural environment, such as temperature, humidity, pollution level, etc. Energy efficiency is one of the most crucial design criteria in battery-powered WSNs in challenging applications. Data transmission and reception, in particular, waste a lot of energy. As a result, the radio transmission of WSNs can be managed to achieve higher energy efficiency^1^. The Table 1 provides the list of acronyms used in the paper.

Unmanned aerial vehicles (UAVs) have been proven to be a promising solution to tackle some of these difficulties, supporting sustainable development goal (SDG) 7 on energy efficiency. UAVs can improve WSNs capabilities by increasing network coverage, enabling effective data aggregation, and improving communication reliability through relay nodes^2^. UAV-assisted WSNs represent a versatile and flexible solution for monitoring and data acquisition in different environments by integrating the benefits of UAVs within WSNs. In UAV assited Wireless Sensor Networks (UWSNs), UAVs directly listen to the sensor nodes for data transmission and considerably reduce the communication between the sensor nodes, therefore saving the energy for listening to the neighbors^3^. The other benefit of utilizing UAVs in WSNs is that there is an open area between the sensor fields and UAVs. The free space will increase the possibility of the signal decaying, which is very significant in sensor-to-BS communication. Moreover, high-level signal processing units and multiple antennas are mounted on UAVs, meaning that some weak signals can also be detected^4^^5^.

**Table 1:** List of Acronyms.

**Acronym**	**Definition**
ABMA	Adaptive BMA
AO-ALOHA	Adaptive-Opportunistic ALOHA
AP-MAC	Advanced Prioritized MAC Protocol
BMA	Bit-Mapping Access
CH	Cluster Heads
CSMA/CA	Carrier Sensing Multiple Access Collision Avoidance
DAABMA	Deviation-Aware Adaptive Bit-Mapping Access
DCFIS	Distributed-Coordinated Inter-Frame Space
E-BMA	Energy Efficient Bit-Mapping Access
EA-TDMA	Energy-Efficient Aggregation TDMA
EC-CMAC	Energy Consumption and Channel Gain Cooperative Medium Access Control
EF-MAC	Energy-Efficient MAC Protocol
FANet	Flying Ad Hoc networks
FD	Full Duplex
HP-MAC	Hybrid MAC Protocol
IoT	Internet of Things
MAC	Medium Access Control
PDR	Packet Delivery Ratio
PF-MAC	Priority Aware Fast MAC Protocol
TDMA	Time Division Multiple Access
UAV	Unmanned Aerial Vehicle
UWSN	UAV-Assisted Wireless Sensor Networks
WSN	Wireless Sensor Network

In addition, numerous efforts are made to enhance energy consumption when using UAV or Flying Ad Hoc networks (FANet) for various scenarios, i.e., EF-MAC^6^, MAC-priority handling^7^, FD-MAC^8^, EA-TDMA and BMA^9^, and EC-CMAC^10^. Despite these advancements, there is still a necessity to further reduce energy consumption to extend the battery life of devices involved in communication. Additionally, the authors in^11^ have performed experiments with EA-TDMA, TDMA, BMA, and E-BMA to prove the superiority of the E-BMA approach. However, the UAV is not integrated to further improve the performance for large WSN and mission-critical applications. Therefore, in the present work, Energy Efficient UAV-assisted Two-Layer Hierarchical (EE-UAV-TLH) MAC is proposed to further improve the performance. The contributions and novelty aspects of the proposed work are discussed below:

- A two-layer hierarchical mathematical model is introduced for analyzing energy efficiency within TDMA, ETDMA, and BMA frameworks.
- Comprehensive energy consumption analysis is conducted, covering transmission, reception, idle state, sleep state, and event generation probability by varying numbers of sensor nodes, cells, and other parameters.
- A Bit-Mapping Access approach is integrated to enhance the energy efficiency by transmitting data to cluster heads only when necessary, thereby reducing energy consumption from unnecessary transmissions.

## Related studies and background

Various MAC protocols are reviewed for different application-specific WSNs. The literature study is divided into various categories, i.e., hybrid MAC, UAV-assisted MAC, energy-efficient MAC, priority-assisted MAC etc.

Sedat et al.^12^ developed a unique hybrid MAC protocol named LD-HMAC to serve data acquisition requests in dense IoT networks. The hybrid protocol merges TDMA with CSMA to create enhanced operational capabilities. The investigation confirms that LD-HMAC outperforms standard time-sensitive IoT protocols when examining network utilization along with end-to-end latency. Rusyadi et al.^13^ proposed the HP-MAC protocol for data acquisition in wireless sensor networks by unmanned air vehicles, which combines CSMA/CA and TDMA mechanisms. The protocol provides impartiality to sensor nodes while giving superior performance in terms of network throughput and packet delivery ratio (PDR). Vanitha et al.^5^ proposed a new hybrid MAC protocol to increase the fair data transfer rate for both the opposing nodes in unmanned aerial vehicle (UAV)-based WSNs. HP-MAC is designed to improve effective node detection during data collection by integrating CSMA/CA and TDMA into the four aforementioned time slots. This method proposed better throughput, packet delivery ratio (PDR), and average time delay, but also provided direct research recommendations regarding energy analysis and multi-UAV scenarios.

Shreya et al.^14^ introduced a novel priority-aware fast MAC (PF-MAC) protocol designed for industrial IoT (IIoT) systems, with the assistance of UAVs. There is a protocol designed to share data quickly and reliably. PF-MAC was reported to hold the maximum performance and offers improved energy conservation with increased throughput and lower latency than other protocols. Williams et al.^15^ developed a prioritized MAC protocol to perform data collection of industrial wireless sensor networks (IWSNs) using unmanned aerial vehicles (UAVs). The study designed a level-siding access channel while utilizing Distributed-Coordinated Inter-Frame Space(DCFIS) to reduce delay time for high-priority nodes and ensure priority access to the owned ground nodes for the access channel. The evaluation revealed that the new protocol can achieve both higher levels of network fairness tools and delivery packet ratios while reducing message delays when compared to the CSMA/CA models. Sabitri et al.^6^ introduced the Energy-Efficient MAC Protocol(EF-MAC), an energy-efficient high-speed MAC protocol tailored for UAV-assisted wireless sensor networks (UWSNs). This protocol addresses critical needs such as border monitoring and disaster management. By integrating variable-slot TDMA registers for data transmission with CSMA for node enrollment, the design optimizes energy usage and minimizes communication delay. Both simulation and analytical model results demonstrated that EF-MAC is more energy-efficient and exhibits lower latency. ELASTIC is the platform that the authors propose as the next step in the evolution of UAV-aided WSNs, and it resolves both energy efficiency and channel contention regulatory issues. The platform utilizes MAC-priority handling for WGS channels competing with tracking transmission opportunities and traffic levels, along with an Advanced Wake Radio system for UAV detection by WGSs. The study achieved these results through experimentation on congested WSN environments and found that the system increased throughput by over 50% relative to classical CSMA/CA mechanisms^7^.

Tae-Yoon et al.^8^ proposed an energy-efficient full-duplex (FD) medium access control (MAC) protocol with the goal of improving the energy efficiency and throughput in UAV base-station (UAV-BS) aided wireless communication networks. An analytical model that encompassed the FD pair probability was necessary for their analyses since previous studies did not fully consider this parameter. Simulation results showed that the FD MAC protocol proposed achieved higher throughput and lower energy consumption in the UAV-BS systems. Jiehong et al.^10^ proposed EC-CMAC protocol as an Energy Consumption and Channel Gain Cooperative Medium Access Control solution for Flying Ad Hoc Networks operating in complex environments to enhance network performance. The protocol allows adaptive transmission modes through a relay selection strategy where node energy levels and transmit power estimation provide the foundation. Results from MATLAB 2024b (https://in.mathworks.com/videos/r2024b-release-highlights-1725868702072.html) simulations showed that EC-CMAC outperformed current protocols by extending network operability, improving message transmission rates, and reducing system delays. Ling et al.^16^ developed the Adaptive-Opportunistic Aloha (AO-Aloha) protocol as a bespoke cross-layer media access control approach for UAV-wireless sensor networks. The protocol achieves more efficient energy management through UAV beacon signals that activate operators into low-power sleep while also applying a priority-based channel allocation system to prevent conflicts. The simulation evidence shows that AO-Aloha means better overall throughput by more than 30% than Observant Aloha currently implements. Shafiullah et al.^11^ developed E-BMA as a new energy-saving MAC protocol specifically constructed for vehicle health monitoring applications within railway WSN environments. Energy performance improves for low and medium traffic rates through E-BMA because it minimizes the time when devices are inactive during contention periods. Analytical and simulation data showed that E-BMA performed better than previous protocols, including EA-TDMA, TDMA, and BMA. Performance evaluation results show that E-BMA delivers better results than other approaches when monitoring low to medium-vehicle traffic. Tolani et al.^1^ developed Deviation-Aware Adaptive Bit-Mapping Access (DAABMA) as a protocol that enhances energy efficiency through data transmission optimization alongside redundancy minimization of WSN. The transmission method provides efficient and standardized data transfers through adaptive segment distribution informed by sensor data sensitivity values, combined with deviation calculation and bit mapping. DAABMA delivered energy-efficient operations through extracted savings of 11% to 42% relative to EBMA while surpassing ABMA by 20% to 28%. Test results demonstrate that DAABMA functions well as a solution for wireless sensor network applications limited by energy constraints. Philipose and Rajesh^17^ present a new energy-efficient MAC protocol named Time Adaptive Bit Map Assisted (TA-BMA) protocol for WSN sensors in a railway monitoring system. The work concentrates on minimizing the energy consumption of sensor nodes installed inside mobile railway wagons with regard to clustering and an adaptive time-slot scheduling. TA-BMA enables nodes to go back to sleep after transmitting the data to mitigate idle listening. Simulation results obtained by the QualNet simulator demonstrate that TA-BMA outperforms EA-TDMA and E-BMA protocols in terms of energy consumption and throughput during light to medium traffic conditions.

**Table 2:** Comparative overview of various MAC protocols.

**Protocol**	**Year**	**F**	**PDR**	**THR**	**DLY**	**EFF**	**NU**	**PERF**	**CONT**	**CA**	**DO**
Hybrid MAC (CSMA/CA + TDMA)^13^	2021	✓	✓	✓	✗	✗	✗	✗	✗	✗	✗
Priority-based MAC (CSMA/CA)^14^	2021	✓		✓	✓	✓	✗	✗	✗	✗	✗
Hybrid MAC (TDMA + CSMA)^12^	2024	✗	✗	✗	✓	✗	✓	✗	✗	✗	✗
UAV-assisted MAC (Modified CSMA/CA)^4^	2020	✗	✗	✗	✗	✗	✓	✗	✗	✗	✗
Priority-based MAC (CSMA/CA)^15^	2021	✓	✓	✓	✗	✗	✗	✗	✗	✗	✗
Hybrid MAC (CSMA + TDMA)^6^	2020	✗	✗	✗	✓	✓	✗	✗	✗	✗	✗
Full-duplex MAC^8^	2023	✗	✓	✓	✗	✓	✗	✗	✗	✗	✗
Opportunistic Network Coded Cooperation (TDMA)^18^	2023	✗	✓	✗	✓	✗	✗	✗	✓	✗	✗
Priority-based MAC with Active Wake-up Radio^7^	2020	✗	✗	✗	✓	✓	✗	✗	✓	✗	✗
Advanced Prioritized MAC (AP-MAC)^19^	2016	✗	✗	✗	✗	✗	✗	✗	✗	✗	✗
Firefly Optimization-based MAC^20^	2019	✗	✗	✗	✗	✗	✓	✗	✗	✓	✗
Hybrid MAC (CSMA/CA + TDMA)^5^	2021	✓	✓	✓	✓	✓	✗	✗	✗	✗	✗
Energy-efficient MAC^10^	2024	✗	✗	✗	✗	✗	✗	✗	✗	✗	✗
Priority-driven frame selection and routing^21^	2016	✗	✗	✗	✗	✗	✗	✗	✓	✗	✗
Cross-layer MAC (Opportunistic Aloha)^16^	2016	✗	✗	✗	✗	✗	✗	✗	✗	✗	✗
Hybrid MAC (TDMA + CSMA)^22^	2010	✗	✗	✗	✗	✗	✗	✗	✗	✗	✗
Markov Chain-based MAC (IEEE 802.15.4)^23^	2021	✗	✗	✗	✗	✗	✗	✗	✗	✗	✗
Energy-efficient MAC^11^	2013	✗	✗	✗	✗	✗	✗	✗	✗	✗	✗
EA-TDMA + BMA (Energy-efficient aggregation)^9^	2018	✗	✗	✗	✗	✗	✗	✗	✗	✗	✗
Adaptive Bit-Mapping Access^1^	2024	✗	✗	✗	✗	✗	✗	✗	✗	✗	✓
BMA, ETDMA AND TDMA^24^	2007	✗	✗	✗	✓	✓	✗	✗	✗	✗	✓
Pre-emptive-Resume Priority (PRP) MAC^25^	2024	✓	✗	✗	✓	✓	✗	✗	✗	✗	✗
LEACH (Low-Energy Adaptive Clustering Hierarchy),TDMA^26^	2002	✓	✗	✓	✓	✓	✗	✗	✗	✗	✓
ECM-MAC,SMAC^27^	2021	✗	✗	✗	✓	✓	✗	✗	✓	✗	✗
Time Adaptive Bit Map Assisted (TA-BMA)^17^	2016	✗	✗	✓	✓	✓	✗	✗	✗	✗	✗
Multi-access Edge Computing (MEC),CSMA/CA based MAC^28^	2024	✗	✗	✓	✗	✓	✗	✗	✗	✗	✗
CSMA-based MAC protocol-UD-MAC^29^	2023	✗	✗	✓	✓	✗	✗	✗	✗	✗	✓

Note: **F** = Fairness, **PDR** = Packet Delivery Ratio, **THR** = Throughput, **DLY** = Delay, **EFF** = Energy Efficiency, **NU** = Network Utilization, **PERF** = Performance, **CONT** = Channel Contention, **CA** = Collision Avoidance, **DO** = Data Optimization.

The research team in^20^ developed a new Medium Access Control strategy specifically designed for Unmanned Aerial Vehicle networks. These approaches use the firefly optimization algorithm to create location-aware timing slots, improving energy efficiency. The approach enhances service quality through improvements that resolve congestion-related and collision-based problems. The simulated results demonstrate significant energy savings at 80.1% alongside reduced packet drops, along with higher throughput and latency minimization (up to 59.04%) and enhanced signal quality. The proposed MAC model demonstrates channel utilization that exceeds 90% when compared to existing approaches. As part of their data collection effectiveness enhancement, the authors in^21^ developed an unmanned aerial vehicle (UAV) supported wireless sensor network data acquisition framework. To maximize data transfer efficiency, the researchers developed a priority-based frame selection method that relied on the geographical position of the sensor node to determine variable transmission speed levels. Simulation results verify that the proposed framework outperforms the state-of-the-art methods while enhancing both network efficiency and energy efficiency. Chen et al.^28^ introduce a UAV-enabled Multi-access Edge Computing (MEC) offloading policy which jointly minimises the energy consumption and the IoT devices’ Age of Information (AoI) measured in terms of the Peak AoI.

A Beta distribution-based traffic model and a CSMA/CA-based MAC model are developed to model real contention and bursty data traffic. Offloading, transmission power, and cache utilization are jointly optimized under the umbrella of DDQN. Simulation results demonstrate that the proposed scheme offers a substantial performance gain as compared to conventional game-theoretical and random offloading designs and provides a better tradeoff between the number of AoI violation events and energy consumption, especially in large-scale device networks.

The authors in^9^ designed Two-Layer Hierarchical Aggregation (TLHA) to extend the wireless sensor network’s (WSN) operational longevity by executing energy-optimized redundant data filtering. A straightforward aggregation algorithm within the protocol applies k-means clustering to distribute and reorganize detected data, which facilitates data filtration at sensor nodes along with cluster heads. Energy Efficient Time Division Multiple Access (EA-TDMA) and Bit-Map-Assisted (BMA) MAC protocols in the TLHA lead to substantial energy consumption cuts. Experimental data shows that TLHA delivers better performance than STCA and DAWF through reduced energy utilization, which reaches 73% less than regular non-aggregated data exchanges. Georgios Y et al.^24^ proposed a BMA MAC protocol, which is designed for cluster-based wireless sensor networks and targets efficiency of energy consumption during event-driven sensing. BMA reduces energy consumption on idle listening and collisions by shifting communications schedules based on real data events. It uses a very simple 1-bit control signalling method during a contention period to determine whether the nodes are idle, then follows with a scheduled data transmission phase. This work proposes two analytical energy models that are verified through ns-2 simulations. From the comparisons in performance, BMA is better than traditional TDMA and energy TDMA (E-TDMA) for both low and moderate traffic in packet delay and energy efficiency.

As discussed and analyzed in the above studies (represented in Table 2), the available MAC protocols for UAV-assisted WSNs do not always efficiently control energy consumption and the transmissions of critical data in a multilayer network. Most recent approaches lack an optimized access mechanism that balances energy efficiency with timely data delivery, especially in mission-critical applications. The proposed Energy Efficient UAV-assisted Two Layer Hierarchical Bit-Mapping Access based MAC protocol aims to bridge these challenges by integrating UAV. The proposed method presents a layered architecture and bit-mapping access scheme to improve the energy conservation, prioritization, and bandwidth utilization.

## Mathematical model and analysis

**Fig. 1:**
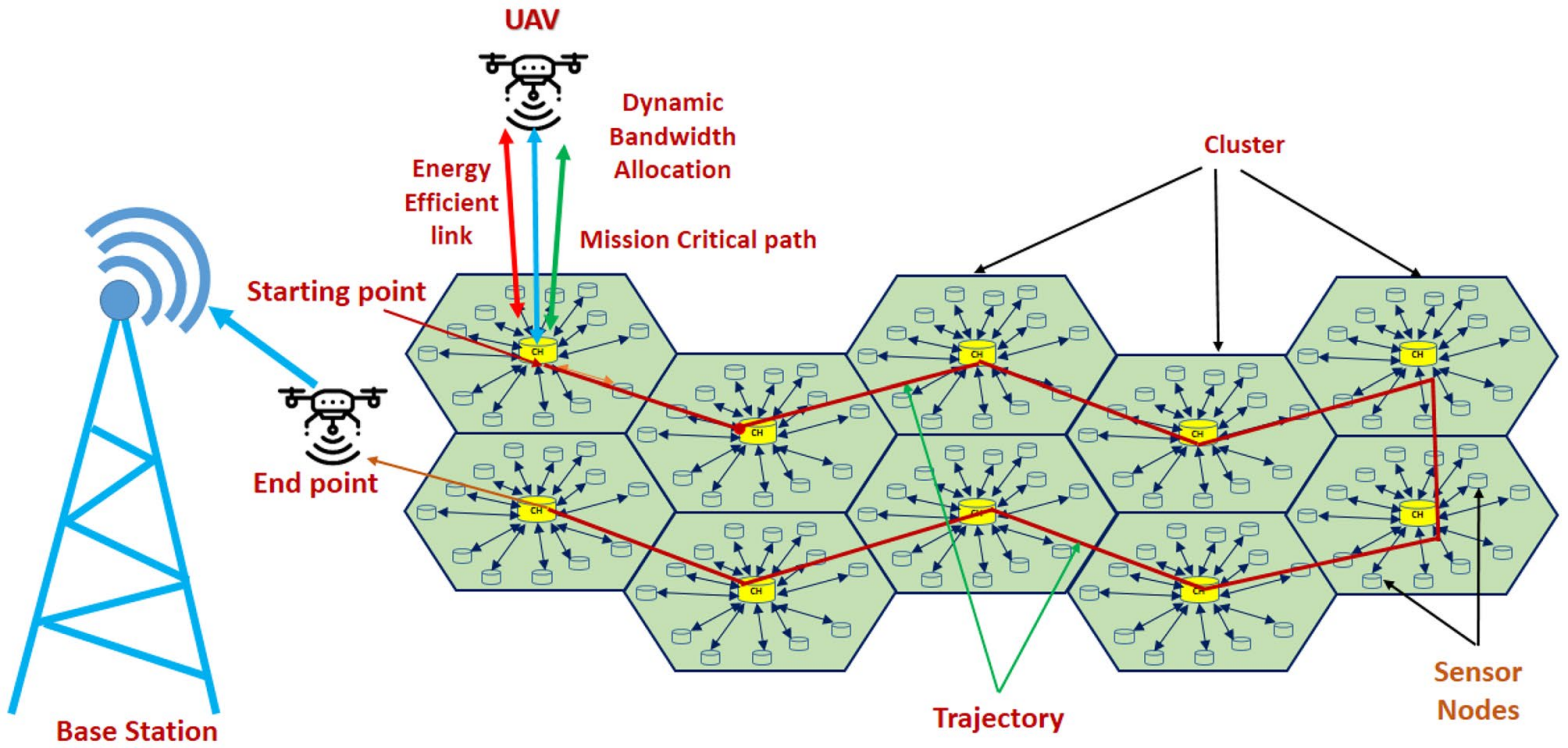
Architecture of the proposed WSN-UAV network.

The proposed energy consumption model is structured into two hierarchical layers. The architecture model of the proposed method is shown in Fig. 1. In practical deployments, sensor nodes are strategically positioned around a designated Cluster Head (CH) to monitor and collect data from specific regions around it. Each sensor node is assigned to a particular CH, as illustrated in Fig. 1. In the first layer, sensor nodes (SNs) transmit data to their respective cluster head (CH) nodes. In the second layer, CH nodes forward the aggregated data to the UAV node. The UAV then performs further aggregation and transmits the collected data to the base station (BS). This model focuses solely on the transceiver power, considering only the transmission and reception energy for evaluating overall energy consumption. UAV lifting power consumption is not considered in the model as the primary focus is on evaluating communication efficiency at the MAC layer, where transmission and reception energy dominate overall consumption. Including lifting energy, which is typically constant and unrelated to MAC operations, would shift the scope toward UAV dynamics rather than network protocol performance. The equation-wise energy consumption model is discussed below.

### Two layer hierarchical TDMA

In this scenario, the Two-Layer Hierarchical TDMA model is considered for Energy consumption analysis. In this approach, the system is made of cell-based sensor nodes (SNs), and the SNs are arranged in clusters with a Cluster Head (CH) that collects data from the same SNs. The CHs subsequently send the collected data to a UAV, which forwards the information to the Base Station (BS). Thus, the overall energy consumption occurs at three distinct stages: SN to CH communication, CH to UAV communication, and UAV to BS communication. The frame architecture and mathematical model are discussed in the next section.

#### Frame architecture of two layer hierarchical TDMA

**Fig. 2:**
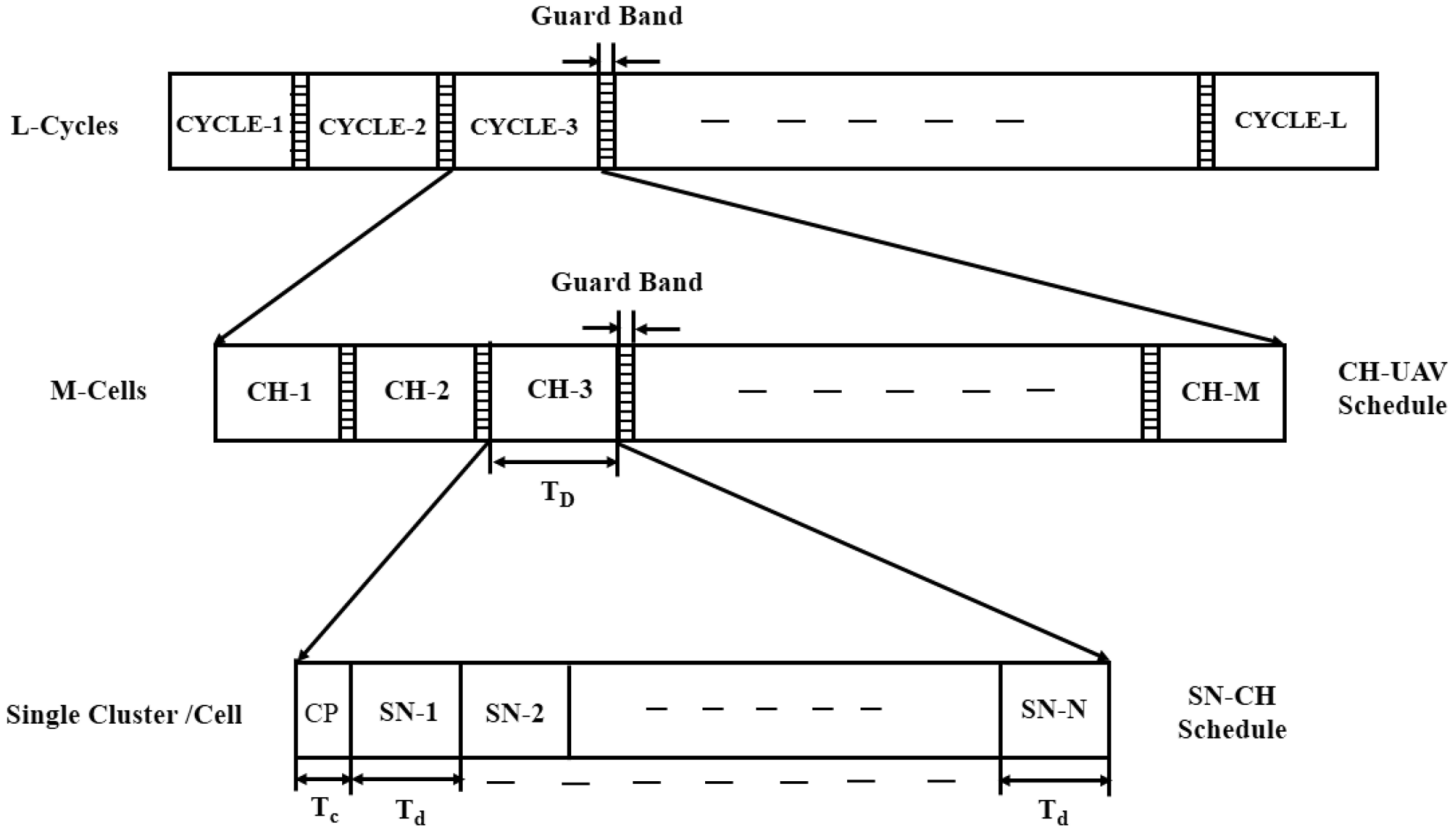
TDMA frame architecture for WSN-UAV network.

The frame architecture of the traditional TDMA method for a two-layer hierarchical model is shown in Fig. 2. During the contention period, each Cluster Head (CH) initiates the communication frame by broadcasting a control packet. This packet serves to synchronize the network and allocate dedicated time slots to all associated sensor nodes. Once the slots are assigned, the sensor nodes transmit their sensed data in accordance with their designated Time Division Multiple Access (TDMA) slots, ensuring collision-free and energy-efficient communication. At the sensor node level, the SN transmits data to its CH during its allotted TDMA timeslot, thereby consuming transmission power. Idle nodes consume energy due to idle listening but do not have data to transmit. The energy consumed by various SNs in the cluster constitutes the total energy, which includes transmission energy, reception energy, and idle power consumption. Then CHs process and aggregate the data from their corresponding SNs and forward it to the UAV. Such a CH to UAV communication brings an extra energy overhead, such as transmission power to send the control packets, reception power to collect acknowledgment, and idle power for the waiting nodes. The UAV passes the aggregated data from all CHs to BS. The total energy consumed at the network consists of energy consumption from the energy of SN to CH, CH to UAV, and UAV to BS with extra wasted power in idle listening and sleep mode.

#### Mathematical model of two layer hierarchical TDMA

In first case the mathematical model is analyzed for the TDMA framework. The complete network is modeled and analyzed for the two layer hierarchal architecture. The Table 3 represents the individual terms in the equations^1,30^.

**Table 3:** Definition of individual terms in equations for TLH-TDMA.

**Symbol**	**Definition**
$EC_{TLH-TDMA}^{Cont}$	Energy Consumption during contention period for Two Layer Hierarchical TDMA
$EC_{TLH-TDMA}^{DTP}$	Energy Consumption during data transmission period for Two Layer Hierarchical TDMA
$EC_{TLH-TDMA}^{CELL}$	Energy Consumption of a cell/cluster for Two Layer Hierarchical TDMA
$EC_{TLH-TDMA}^{Cycle}$	Energy Consumption during sensor node and cluster head communication for Two Layer Hierarchical TDMA
$EC_{TLH-TDMA}^{CH-UAV}$	Energy Consumption during Cluster head to UAV transmission for Two Layer Hierarchical TDMA
$EC_{TLH-TDMA}^{UAV-BS}$	Energy Consumption during UAV to base station transmission for Two Layer Hierarchical TDMA
$EC_{TLH-TDMA}^{Total}$	Total Energy Consumption during transmission for Two Layer Hierarchical TDMA

The energy consumed by one cell during the contention period is estimated by combining the transmission energy of the cluster head and the reception energy of the N listening sensor nodes for the transmission of control packets. It highlights how both transmitter and receiver activities contribute to overall energy usage during contention^1,30^.

1 $$\begin{aligned} EC_{TLH-TDMA}^{Cont} = P_t \times T_c + N\times P_r \times T_c \end{aligned}$$

Equation 2 represents the total energy consumption during the data transmission phase (DTP) in one TLH-TDMA cell. Here, *p* is the probability of a node transmitting data, $P_t$ and $P_r$ are the transmission and reception powers, respectively, $P_i$ is the idle power, $T_d$ is the duration of the data slot, and *N* is the number of nodes. The first term accounts for the energy consumed in transmission by SN, the second term for the idle energy of non-transmitting nodes (multiplied by 2 to represent both SN and CH), and the third term for the reception energy of CH. Multiplying the entire expression by *N* gives the total DTP energy consumption across all nodes in the cell^1,30^.

2 $$\begin{aligned} EC_{TLH-TDMA}^{DTP} = \bigg (p\times P_t \times T_d+2 \times (1-p) \times P_i \times T_d + p\times P_r \times T_d \bigg )\times N \end{aligned}$$

The total energy consumption is sum of contention access period (represented in Eq. 1) and contention free period (represented in Eq. 2) in single cell can be given by Eq. 3^1,11,30^.

3 $$\begin{aligned} EC_{TLH-TDMA}^{CELL} = P_t \times T_c + N\times P_r \times T_c+ \bigg (p\times P_t \times T_d+2 \times (1-p) \times P_i \times T_d + p\times P_r \times T_d \bigg )\times N \end{aligned}$$

Equation 4 represents the total energy consumption for a complete transmission cycle across all *M* cells in the TLH-TDMA protocol. The term $EC_{TLH-TDMA}^{CELL}$ denotes the energy consumed in one active cell during its contention and data transmission phases. The second term, $(M-1)\times (N\times T_d+T_c)\times N \times P_s$, accounts for the sleep energy consumed by nodes in the remaining $(M-1)$ inactive cells while one cell is active. Here, $T_d$ is the data transmission duration, $T_c$ is the control packet duration, and $P_s$ is the power consumed in sleep mode. The entire expression is multiplied by *M* to represent the total energy consumed over one complete cycle involving all *M* cells in the network^1,30^.

4 $$\begin{aligned} EC_{TLH-TDMA}^{Cycle} = \bigg (EC_{TLH-TDMA}^{CELL} + (M-1)\times (N\times T_d+T_c)\times N \times P_s\bigg )\times M \end{aligned}$$

Equation 5 represents the energy consumption during communication between the CHs and the UAV in the TLH-TDMA protocol. The first term accounts for the transmission energy by active CHs, the second term captures the idle energy consumed by non-transmitting CHs (including state transitions), and the third term represents the reception energy by the UAV. The entire expression is multiplied by *M*, the total number of CHs (one per cell), to obtain the total energy consumption for CH-to-UAV communication across all cells^1,30^.

5 $$\begin{aligned} EC_{TLH-TDMA}^{CH-UAV} = \bigg (p\times P_t \times T_D+2 \times (1-p) \times P_i \times T_D + p\times P_r \times T_D \bigg )\times M \end{aligned}$$

Equation 6 represents the energy consumption during the communication phase between the UAV and the BS in the TLH-TDMA protocol. Since the UAV transmits aggregated data from each of the *M* cluster heads to the BS, the total energy consumption is calculated as $M \times P_t \times T_D$, assuming one transmission per cluster’s data^1,11,30^.

6 $$\begin{aligned} EC_{TLH-TDMA}^{UAV-BS} = M\times P_t \times T_D \end{aligned}$$

The total energy consumption for Two-Layer Hierarchical TDMA for overall L Cycles can be estimated as a sum of energy consumptions of two layers, i.e. SN-CH and CH-UAV, and UAV-BS as well after each cycle as given in Eq 7^1,30^.

7 $$\begin{aligned} EC_{TLH-TDMA}^{Total} = \Bigg (EC_{TLH-TDMA}^{Cycle}+EC_{TLH-TDMA}^{CH-UAV}+EC_{TLH-TDMA}^{UAV-BS}\Bigg ) \times L \end{aligned}$$

### Two layer hierarchical E-TDMA

In this scenario, the Two-Layer Hierarchical Energy-aware TDMA model is considered for Energy consumption analysis. ETDMA incorporates energy-saving mechanisms in addition to common TDMA by enabling SNs to deactivate their radios when they do not have data to transmit, thus minimizing idle listening and reducing energy consumption accordingly.

#### Frame architecture of two layer hierarchical ETDMA

**Fig. 3:**
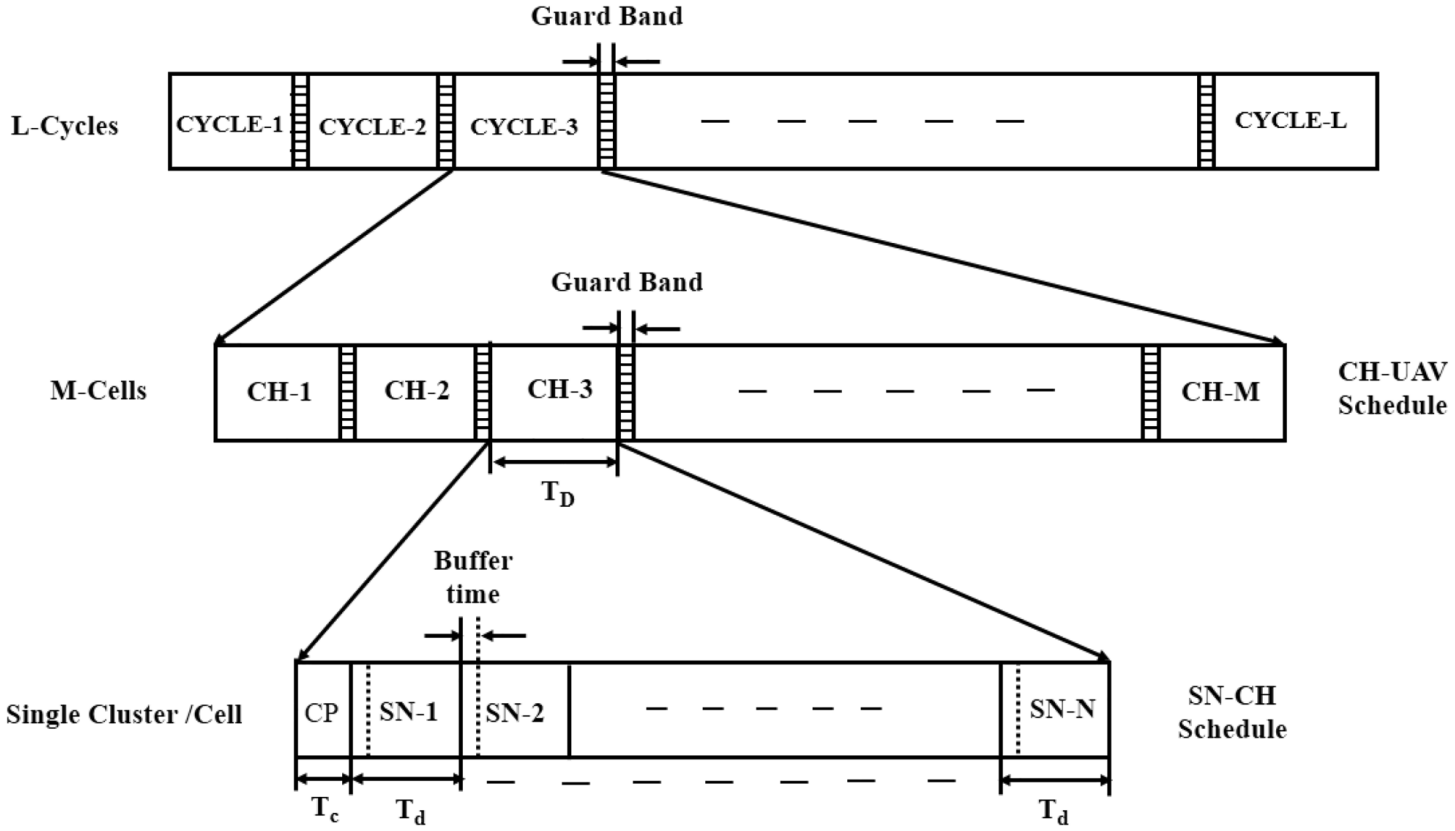
ETDMA frame architecture for WSN-UAV network.

The frame architecture of the ETDMA method for a two-layer hierarchical model is shown in Fig. 3. At the sensor node level, all SNs share the same time-division schedule for sending data to their CH. The energy consumed in this period is the sum of transmission energy (to transmit data packets), reception energy (to receive control packets), and idle energy consumption. ETDMA implements an energy-saving sleep mode that enables idle nodes to consume much less energy than idle listening mode as in traditional TDMA. Overall, energy consumption can be minimized, especially in low-traffic situations where many nodes have no packets to transmit. As the CHs gather their data from associated SNs, they aggregate and send it to the UAV. The second phase of CH-to-UAV communication involves control packet transmission, reception acknowledgment, and data packet transmission. The major improvement in ETDMA is to let the CHs enter sleep mode, avoiding idle mode when CH has no data to send, which alleviates the power consumption caused by unnecessary idle nodes^31^. The UAV transmits the aggregated data to the Base Station (BS) after collecting data from the CHs. The energy consumed in ETDMA is the summation of energy consumed at the SN level, CH level, and UAV level.

#### Mathematical model of two layer hierarchical ETDMA

In the second case, the mathematical model is developed for the ETDMA (Energy-aware TDMA) framework. The analysis covers the complete network operation within a two-layer hierarchical architecture. In this architecture, the first layer manages communication between sensor nodes and their respective cluster heads, while the second layer handles data transmission from CHs to the UAV, and subsequently from the UAV to the base station. The Table 4 represents the individual terms in the equations below:

**Table 4:** Definition of individual terms in equations for TLH-ETDMA.

**Symbol**	**Definition**
$EC_{TLH-ETDMA}^{Cont}$	Energy Consumption during contention period for Two Layer Hierarchical ETDMA
$EC_{TLH-ETDMA}^{DTP}$	Energy Consumption during data transmission period for Two Layer Hierarchical ETDMA
$EC_{TLH-ETDMA}^{CELL}$	Energy Consumption of a cell/cluster for Two Layer Hierarchical ETDMA
$EC_{TLH-ETDMA}^{Cycle}$	Energy Consumption during sensor node and cluster head communication for Two Layer Hierarchical ETDMA
$EC_{TLH-ETDMA}^{CH-UAV}$	Energy Consumption during Cluster head to UAV transmission for Two Layer Hierarchical ETDMA
$EC_{TLH-ETDMA}^{UAV-BS}$	Energy Consumption during UAV to base station transmission for Two Layer Hierarchical ETDMA
$EC_{TLH-ETDMA}^{Total}$	Total Energy Consumption during transmission for Two Layer Hierarchical ETDMA

The equations 8, 9, and 10 describe the energy consumption components within a single cell of the proposed TLH-ETDMA framework. Equation 8 represents the energy consumption during the **contention period** in a single TLH-ETDMA cell^1,11,30^.

8 $$\begin{aligned} EC_{TLH-ETDMA}^{Cont} = P_t \times T_c + N\times P_r \times T_c \end{aligned}$$

Equation 9 models the energy consumed during the data transmission phase (DTP). Here, *p* is the probability of a node transmitting data. The first term, $p \times P_t \times T_d$, denotes the energy used for transmission. The second term, $(1-p) \times P_i \times T_d$, accounts for the idle energy of non-transmitting nodes. The third term, $(1-p) \times P_e \times T_e$, captures the energy spent by non-source nodes for buffer checking. The last term, $p \times P_r \times T_d$, represents the energy consumed by nodes receiving the transmitted data. The entire expression is multiplied by *N* to obtain the total energy consumption for all nodes during DTP^1,11,30^.

9 $$\begin{aligned} EC_{TLH-ETDMA}^{DTP} = \bigg (p\times P_t \times T_d+ (1-p) \times P_i \times T_d+(1-p) \times P_e \times T_e + p\times P_r \times T_d \bigg )\times N \end{aligned}$$

Equation 10 gives the total energy consumption in a TLH-ETDMA cell, by summing the energy used in the contention period and the data transmission phase. It provides a complete picture of the communication-related energy cost within a single cell of the hierarchical ETDMA framework^1,30^.

10 $$\begin{aligned} EC_{TLH-ETDMA}^{CELL} = EC_{TLH-ETDMA}^{Cont}+EC_{TLH-ETDMA}^{DTP} \end{aligned}$$

Equation 11 models the total energy consumption for a complete communication cycle across all *M* cells in the TLH-ETDMA network. The term $EC_{TLH-ETDMA}^{CELL}$ denotes the energy consumed in one active cell, which includes both the contention and data transmission phases. The second part, $(M-1)\times (N\times T_d + T_c)\times N \times P_s$, calculates the energy consumed by the nodes in the remaining $(M-1)$ inactive cells while they are in sleep mode during the operation of the active cell^1,11,30^.

11 $$\begin{aligned} EC_{TLH-ETDMA}^{Cycle} = \bigg (EC_{TLH-TDMA}^{CELL} + (M-1)\times (N\times T_d+T_c)\times N \times P_s\bigg )\times M \end{aligned}$$

Equation 12 represents the energy consumption during the communication between the CHs and the UAV in the TLH-ETDMA protocol. Here, $P_e$ is the power used for energy estimation over a duration $T_e$. The first term captures the energy used by CHs that transmit data, the second terms account for the idle power consumed by non-transmitting CHs, the third term represents energy consumption of non-source CHs, and the final term represents the energy used by the UAV in receiving data. The entire expression is multiplied by *M*, the total number of CHs (one per cell), to calculate the overall energy consumption for CH-UAV communication across the network^1,11,30^.

12 $$\begin{aligned} EC_{TLH-ETDMA}^{CH-UAV} = \bigg (p\times P_t \times T_D+ (1-p) \times P_i \times T_D + (1-p) \times P_e \times T_e + p\times P_r \times T_D \bigg )\times M \end{aligned}$$

Equation 13 represents the energy consumption during the communication phase between the UAV and the BS in the TLH-ETDMA protocol. Since the UAV transmits aggregated data from each of the *M* cluster heads to the BS, the total energy consumption is calculated as $M \times P_t \times T_D$, assuming one transmission per cluster’s data^1,11,30^.

13 $$\begin{aligned} EC_{TLH-ETDMA}^{UAV-BS} = M\times P_t \times T_D \end{aligned}$$

The total energy consumption for Two-Layer Hierarchical ETDMA for overall L Cycles can be estimated as a sum of energy consumptions of two layers, i.e. SN-CH and CH-UAV, and UAV-BS as well after each cycle as given in Eq. 14^1,30^.

14 $$\begin{aligned} EC_{TLH-ETDMA}^{Total} = \Bigg (EC_{TLH-ETDMA}^{Cycle}+EC_{TLH-ETDMA}^{CH-UAV}+EC_{TLH-ETDMA}^{UAV-BS}\Bigg ) \times L \end{aligned}$$

### Two layer hierarchical BMA

Two-Layer BMA model is implemented where sensor nodes (SNs) are grouped into clusters, anda Cluster Head (CH) collects data from its SNs. Rather than using a fixed-time division schedule (e.g. TDMA), transmission slots are dynamically assigned here based on data present in BMA. This enables nodes to broadcast when needed, minimizing energy waste due to idle listening and collision. Every CH forwards the information aggregated from SNs to a UAV that transmits it to a Base Station (BS) for processing.

#### Frame architecture of two layer hierarchical BMA

**Fig. 4:**
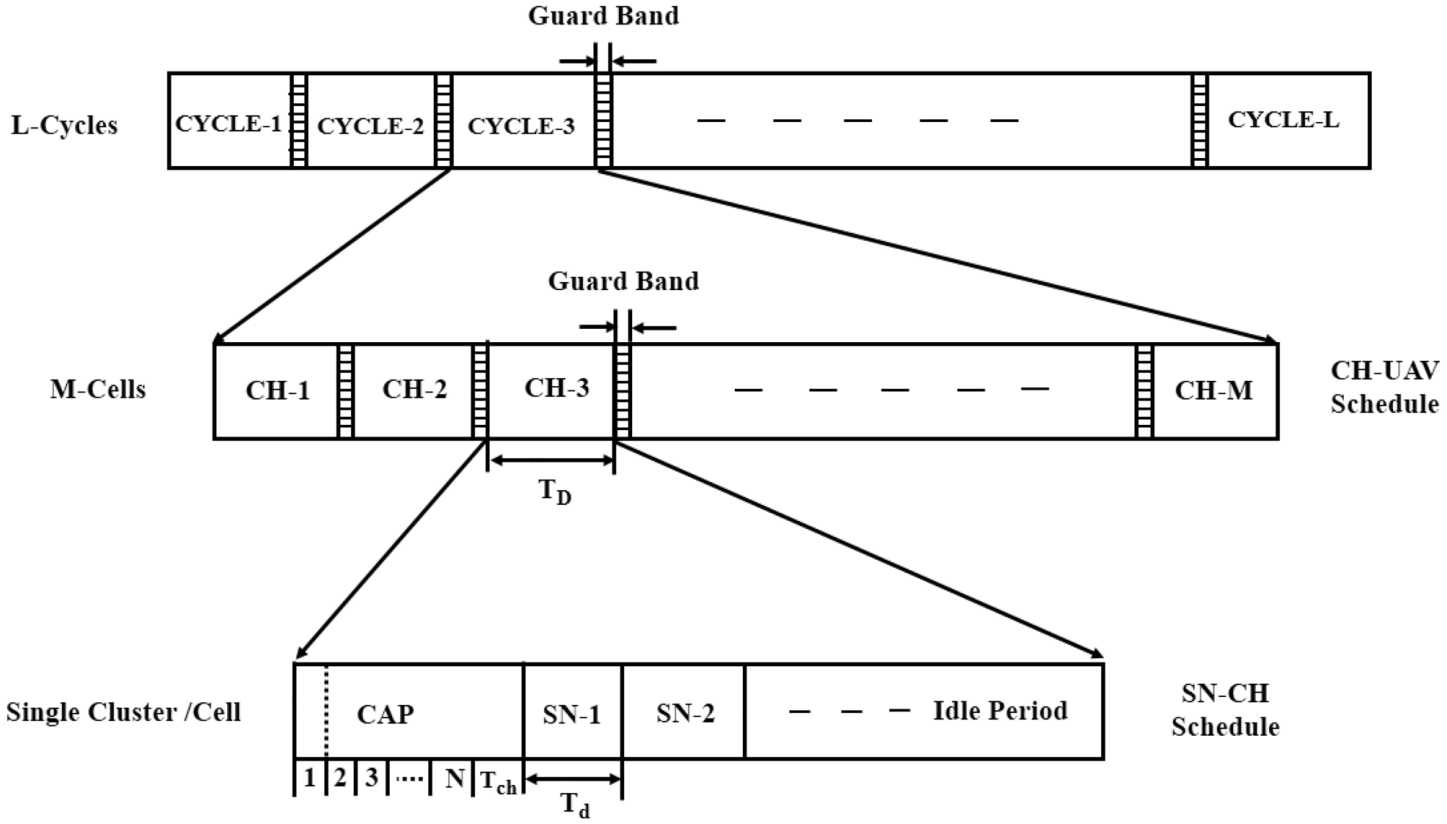
Bit mapping frame architecture for WSN-UAV network.

The frame architecture of the BMA method for a two-layer hierarchical model is shown in Fig. 4. Each SN sends a 1-bit control message during the contention phase to inform the CH of the presence or absence of data to transmit. If no data belongs to a node, it stays in sleep mode to save power. Energy consumption for this phase is the sum of the energy used by the transmit antenna for control packets, idle listening, and receiving power. Then, the nodes with successful transmission will be assigned a transmission slot to send their data to the CH. In comparison to TDMA and ETDMA, BMA saves a great number of unnecessary transmissions by permitting only active nodes to claim the slots in the traffic, especially in low to medium traffic conditions. After collection, the CH collects the information of its corresponding SNs and sends it to the UAV. In comparison with TDMA, where the CHs transmit data every fixed amount of time, BMA gives the privilege of sending to the CHs only when the data needs to be sent, thus less energy is consumed in unnecessary transmissions. The total energy consumption of BMA is given by the sum of the energy consumption from each level (SN, CH, and UAV).

#### Mathematical model of two layer hierarchical BMA

In third case, the mathematical model is developed for the bit-mpa-assisted (BMA) framework. The Table 5 represents the individual terms in the equations below:

**Table 5:** Definition of individual terms in equations for TLH-BMA.

**Symbol**	**Definition**
$EC_{TLH-BMA}^{Cont}$	Energy Consumption during contention period for Two Layer Hierarchical BMA
$EC_{TLH-BMA}^{DTP}$	Energy Consumption during data transmission period for Two Layer Hierarchical BMA
$EC_{TLH-BMA}^{CELL}$	Energy Consumption of a cell/cluster for Two Layer Hierarchical BMA
$EC_{TLH-BMA}^{Cycle}$	Energy Consumption during sensor node and cluster head communication for Two Layer Hierarchical BMA
$EC_{TLH-BMA}^{CH-UAV}$	Energy Consumption during Cluster head to UAV transmission for Two Layer Hierarchical BMA
$EC_{TLH-BMA}^{UAV-BS}$	Energy Consumption during UAV to base station transmission for Two Layer Hierarchical BMA
$EC_{TLH-BMA}^{Total}$	Total Energy Consumption during transmission for Two Layer Hierarchical BMA

Equation 15 represents the energy consumption during the contention phase in a single cell of the TLH-BMA protocol. The first term captures the total energy spent by all *N* nodes, considering the transmitting node’s energy $P_t \times T_c$, the idle energy of non-transmitting nodes $(1-p) \times P_i \times T_c$, the idle energy of $(N-1)$ other nodes, and the reception energy $P_r \times T_{ch}$ during the cluster head broadcast slot allotment duration $T_{ch}$. The second term accounts for the energy consumed by all *N* nodes in listening mode, where a fraction *p* of them receive the transmission ($p \times P_r \times T_c$), while the remaining $(1-p)$ remain in idle mode. The final term $P_t \times T_{ch}$ represents the energy used by the broadcasting in transmitting the slot allotment information to the SNs. Together, these components comprehensively reflect the energy dynamics in the contention phase under the BMA protocol^1,11,30^.

15 $$\begin{aligned} EC_{TLH-BMA}^{Cont} =&\Bigg (P_t \times T_c + (1-p) \times P_i \times T_c+ (N-1) \times P_i \times T_c + P_r \times T_{ch} \Bigg ) \times N \\ \nonumber&+ \bigg (p \times P_r \times T_c) + (1-p) \times P_i \times T_c \bigg ) \times N + P_t \times T_{ch} \end{aligned}$$

Equation 16 represents the total energy consumption during the (DTP) in a single TLH-BMA cell. The term $p \times P_t \times T_d$ accounts for the energy consumed by transmitting nodes, while $p \times P_r \times T_d$ reflects the energy used by receiving nodes. The entire expression is multiplied by *N*, the number of nodes in the cell, to compute the total energy consumption for all participating nodes during data transmission.

16 $$\begin{aligned} EC_{TLH-BMA}^{DTP} = \bigg (p\times P_t \times T_d+p\times P_r \times T_d\bigg ) \times N \end{aligned}$$

Equation 17 gives the total energy consumption in a single cell of the TLH-BMA protocol. It is calculated as the sum of energy consumed during the contention phase ($EC_{TLH-BMA}^{Cont}$) and the data transmission phase ($EC_{TLH-BMA}^{DTP}$).

17 $$\begin{aligned} EC_{TLH-BMA}^{CELL} = EC_{TLH-BMA}^{Cont}+EC_{TLH-BMA}^{DTP} \end{aligned}$$

Eq?? calculates the total energy consumption over one complete transmission cycle across all *M* cells in the TLH-BMA protocol. The term $EC_{TLH-BMA}^{CELL}$ represents the energy consumed in one active cell during its contention and data transmission phases. The second term, $(M-1)\times (N\times T_d + T_c)\times N \times P_s$, accounts for the energy consumed by the nodes in the remaining $(M-1)$ inactive cells while in sleep mode^1,11,30^.

18 $$\begin{aligned} EC_{TLH-BMA}^{Cycle} = \bigg (EC_{TLH-BMA}^{CELL} + (M-1)\times (N\times T_d+T_c)\times N \times P_s\bigg )\times M \end{aligned}$$

The second layer hierarchal communication of CH and UAV is based ETDMA approach only where the non-source nodes checks the buffer and turns-off radio. The buffer checking time is $T_e$ and power consumption is $P_e$. The overall energy consumption is given by Eq. 19

19 $$\begin{aligned} EC_{TLH-BMA}^{CH-UAV} = \bigg (p\times P_t \times T_D+ (1-p) \times P_i \times T_D + (1-p) \times P_e \times T_e + p\times P_r \times T_D \bigg )\times M \end{aligned}$$

Equation 20 represents the energy consumption during the communication phase between the UAV and the BS in the TLH-BMA protocol. Since the UAV transmits aggregated data from each of the *M* cluster heads to the BS, the total energy consumption is calculated as $M \times P_t \times T_D$, assuming one transmission per cluster’s data^1,11,30^.

20 $$\begin{aligned} EC_{TLH-BMA}^{UAV-BS} = M\times P_t \times T_D \end{aligned}$$

The total energy consumption for Two-Layer Hierarchical BMA for overall L Cycles can be estimated as a sum of energy consumptions of two layers, i.e. SN-CH and CH-UAV, and UAV-BS as well after each cycle as given in Eq. 21.

21 $$\begin{aligned} EC_{TLH-BMA}^{Total} = \Bigg (EC_{TLH-BMA}^{Cycle}+EC_{TLH-BMA}^{CH-UAV}+EC_{TLH-BMA}^{UAV-BS}\Bigg ) \times L \end{aligned}$$

It is important to note that the developed mathematical model explicitly incorporates MAC overheads and synchronization costs by including the durations of control packets ($T_c$), buffer-check time ($T_e$), and idle/sleep state transitions. These parameters capture the practical costs of coordination and network synchronization, and therefore the reported improvements in energy consumption reflect gains achieved without neglecting operational overheads. Based on above mathematical model, the analytical analysis is developed and analyzed in result analysis and discussion section.

## Performance analysis and discussions

In this section, the performance of the proposed method is analyzed and compared with integration of various frameworks. The analysis provides the energy efficiency of TLH-TDMA, TLH-ETDMA, and TLH-BMA protocols in a WSN-UAV communication model. Analytical models are developed to analyze the energy consumption for transmission of data from SN to CH and CH to BS via UAV with variations in the number of SNs, cells, and other parameters. Simulation is carried out by considering the parameters taken from IEEE 802.15.4 WPAN and CC2420 RF as shown in Table 6. These include a data rate of 250 kbps, packet sizes (100 Bytes for data packet and 5 bytes for control packet), power consumption metrics for various modes and multicell network with 25 cells each containing 100 nodes^11,32^.

**Table 6:** Network parameters and their values^11,32^.

**Parameters**	**Symbol**	**Value**
Data rate	$D_R$	250 kbps
Data packet size	$T_d$	100 Bytes
Control packet size	$T_c$	5 Bytes
Buffer check time	$T_e$	$0.1 \times T_d$
Nodes in cell	$N_i$	100
Power consumption in transmit mode	$P_t$	62.64 mW
Power consumption in receive mode	$P_r$	57.4 mW
Power consumption in idle mode	$P_i$	62.64 mW
Power consumption in sleep mode	$P_s$	72 $\mu$W
Number of Cells	*M*	25

### Scenario 1

**Fig. 5:**
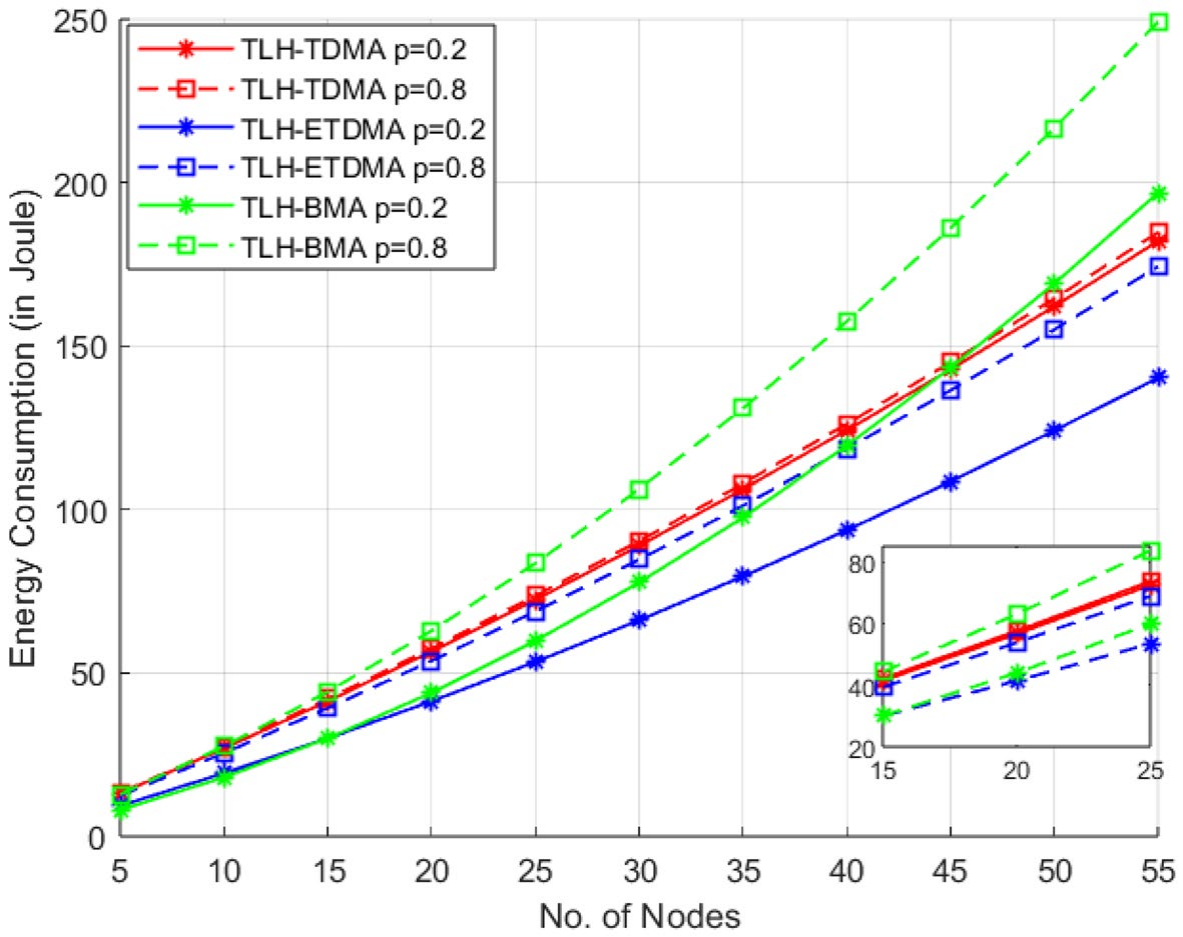
Energy consumption vs sensor nodes case 1.

**Fig. 6:**
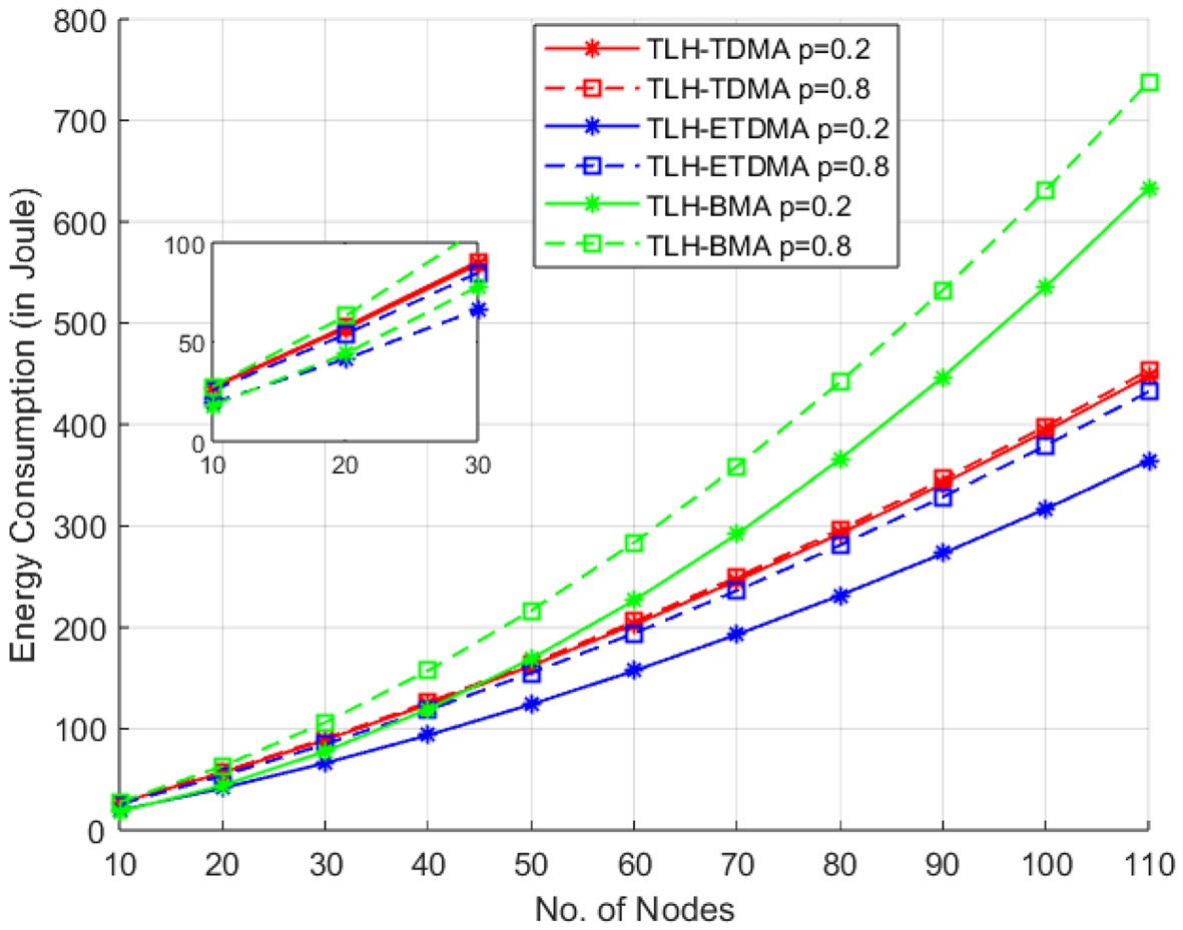
Energy consumption vs sensor nodes case 2.

In this scenario, Figs. 5 and 6 provide the comparison of the energy consumption in integration with three MAC protocol frameworks (TLH-TDMA, TLH-ETDMA and TLH-BMA) and two packet arrival probability extremes, i.e., p = 0.2 and p = 0.8 in TLH architecture consist of sensor nodes, cluster head (CHs) and UAV. In the first graph, the sensor nodes are varied from 5 to 55 nodes while in the second one, nodes are varied from 10 to 110 which provides the insight of scalability from small to large networks. At 55 nodes, TLH-ETDMA achieves 15% savings over TLH-TDMA and 42% over TLH-BMA.

In both Figs. 5 and 6, the energy consumption tends to increase monotonically with the number of nodes, as more data packet communication, control overhead demanded. But when the traffic is denser (p=0.8), all protocols rapidly increase the energy consumption, because the packets are transmitted more frequently. Comparably, TLH-ETDMA delivers better energy efficiency in an consistent manner to different node ranges and under both low and high traffic load conditions. It saves energy well by suppressing idle listening mode, so it is very scalable and reliable in large-scale network and high traffic condition. The TLH-TDMA, on the other hand, has a linear increasing trend of energy consumption, and moderate energy efficiency. It has a superior performance than TLH-BMA in high load, high density of nodes and is worse than ETDMA.

The bit-mapping based MAC protocol TLH-BMA has comparable better performance in low traffic (p = 0.2) and fewer nodes. But its energy consumption increases quickly with node density and increase in traffic due to high overhead traffic in contention period. As the number of nodes increases, bit-mapping duration also increases which causes increase in overhead duration. It implies that the bit-mapping overhead is noticeable when node density and traffic is large. This tendency is enhanced in the second graph (up to 110 nodes in Fig. 6) where TLH-BMA (p = 0.8) presents higher energy equivalent need comparing to the other protocols.

Both figures shows that for 15–30 nodes, in the light network situation, TLH-BMA (p = 0.2) is as good as the TLH-TDMA and is in some times very close to TLH-ETDMA. This indicates that BMA is better operated in the low-traffic, small- scale scenario, therefore there is no need for complex access scheduling. The relative energy efficiency of TLH-ETDMA has a better scalability with the network size and coming next is TLH-TDMA. One of the disadvantages of the TLH-BMA protocol is its poor scalability which could not handle high packet arrival rates and large node density.

### Scenario 2

**Fig. 7:**
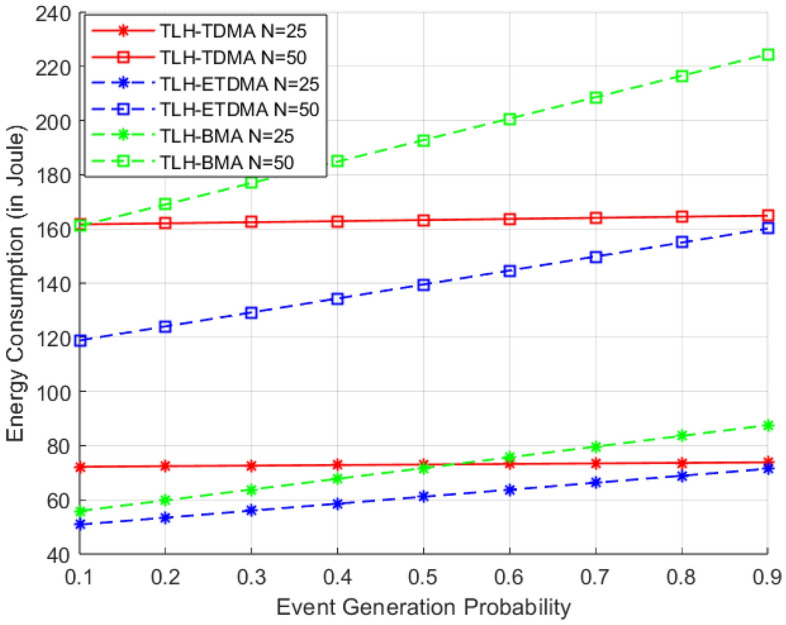
Energy consumption vs event generation probability.

In the Fig. 7, energy consumption is plotted by varying the event generation probability from 0.1 to 0.9 under different MAC frameworks, i.e., TLH-TDMA, TLH-ETDMA, and TLH-BMA in two separate network sizes (N = 25 and 50 nodes). As results show that with increase in the event generation probability, i.e. node to report detecting an event, energy consumption of all MAC protocols also increases. Energy consumption is highly dependent on network size (number of nodes). In all protocols, energy consumption is also higher under N = 50 than N = 25, because of higher amount of data transmission, and transmission overhead. TDMA-based protocols are less dependent on event generation probability, because every node remain in transmit/idle mode in its time slot whether there is an event or not. This results in continuous or quasi-continuous energy consumption. ETDMA presents traffic-aware scheduling that accommodates the energy efficient communication by turning-off radio (sleep mode) when an event does not happen.Therefore, at low probabilities much less energy is consumed. With an increasing probability, more and more events are created and more slots are employed, resulting in an energy increase. This is a behavior that causes ETDMA to be more responsive and scalable, even for N = 50. ETDMA is the most energy efficient protocol for nearly all probabilities, especially at high number of nodes.

For low event generation probability (for example, 0.1–0.3) the TLH-BMA protocol with N = 25 performs even better than TLH-ETDMA. This can be explained by the fact that BMA’s bit-mapped structure can effectively reduce control messages when few events happen. However for larger events the BMA scheme suffers from a high energy consumption of energy overhead, for high probability of collision N = 50, the energy overhead increases rapidly and over TLH-TDMA and TLH-ETDMA at last. This suggests that, though TLH-BMA may be optimal under light traffic density in a sparse network, it does not scale well when there is heavy arrival of events or the network is dense.

### Scenario 3

**Fig. 8:**
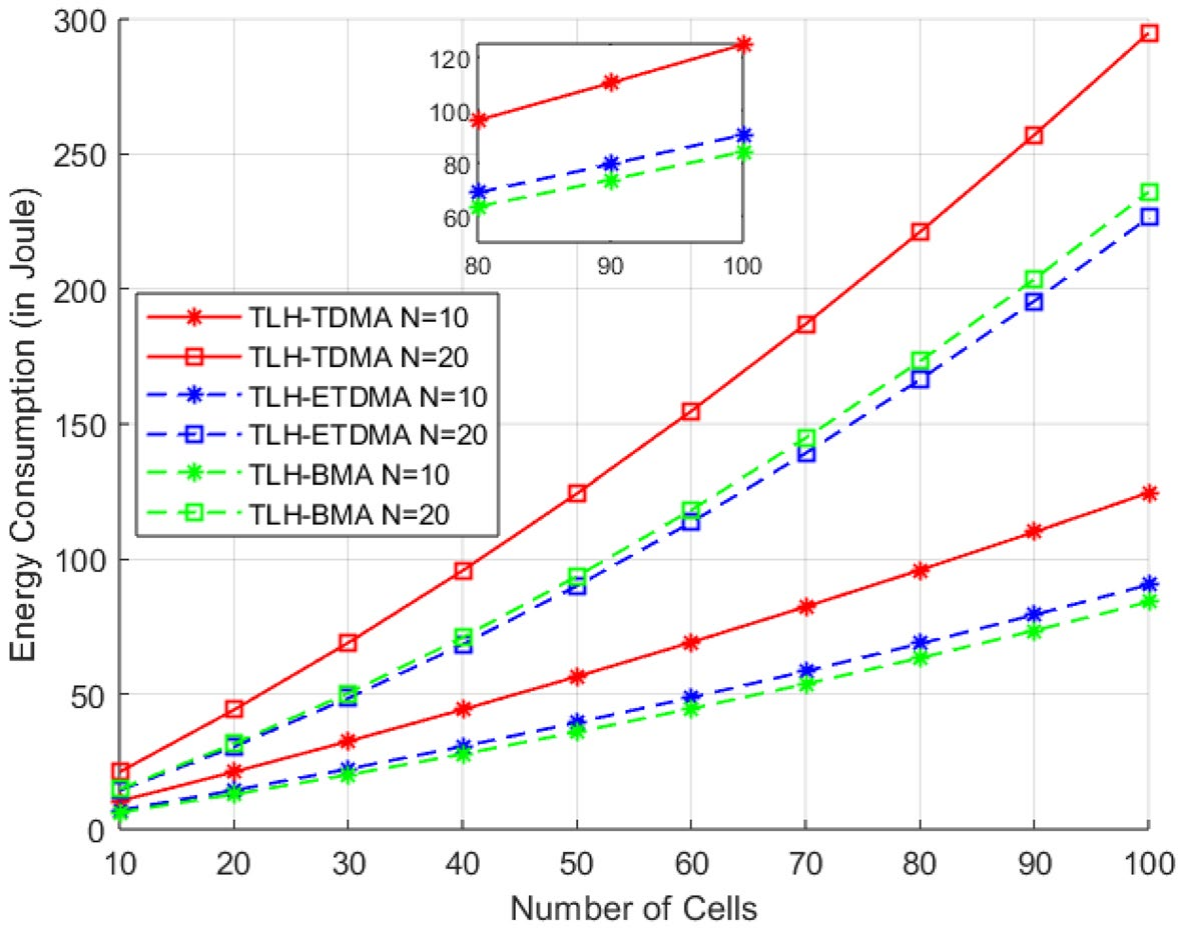
Energy consumption vs no. of cells.

The Fig. 8 analyzes the energy consumption budgets for three MAC frameworks TLH-TDMA, TLH-ETDMA, and TLH-BMA with an increase in number of cells from 10 to 100 under two distinct cases of fixed nodes (10, 20) and a constant rate of event generation of 0.1. This simulation configuration shows how network partitioning through additional cells influences protocol operation in a hierarchical communication model.

From the results we can observe that TLH-TDMA always consumes more energy, in particular when 20 nodes are considered and all cells are in the active mode a greater number of time slots are increasing in all the cells. TDMA rigid time-slot based structure requires all nodes to be active even when event probability is low. The higher the cell size, the higher the number of intra-cluster communications and with TDMA’s static scheduling, the cumulative energy consumption is much higher. On the other hand, TLH-ETDMA outperforms in terms of energy consumption because of its proposed event-based slot allocation. Because of its low event generation probability (0.1), ETDMA can jump the idle slots that exist between and within the cluster through reducing the unnecessary transmission overhead, which is especially useful when the number of cells (and cluster heads) is increased. However, for larger number of nodes, the control and coordination overhead in time slot management increases slightly, which leads to the increase of energy consumption gradually.

However, TLH-BMA exhibits the most energy compact behavior for all the numbers of cells, especially at 10 nodes. A node-specific and bit mapped access is used to find the active node in an accurate manner without having the slot fixed or idle. This effective mapping reduces overhead. At 20 nodes Energy-wise, TLH-BMA has a slightly higher energy consumption than that of under 10 nodes, but it is still better than both TDMA and ETDMA. This is consistent with claims that BMA is extremely light weight in terms of access control, having a low level of coordination overhead, and scales higher with spatial complexity (i.e. more cells). In general, the trend is consistent and shows that as the number of cells increases, inter-cluster transmissions increase, requiring more energy in all protocols. However, the performance of the TLH-BMA in terms of redundant transmissions suppression and resource allocation overhead is the best in case of the under sparse traffic condition and when the sensor network is expanding in space.

**Table 7:** Theoretical comparison of EE-UAV-TLH with recent SOTA MAC protocols.

**Criterion**	**EE-UAV-TLH**	** Wu et al.**^10^, (IEEE TMC)	** Tolani et al.**^1^, (IEEE Access)
Network model	Single-cell clustered WSN with UAV mobile sink	Multi-UAV cooperative FANET	Static-sink WSN with adaptive bit-mapping
Primary objective	Energy-efficient cluster-based MAC with bit-mapping + ETDMA	Energy- and channel-gain-aware cooperative UAV relay selection	Deviation-aware adaptive scheduling for event-driven traffic
Energy consumption (Theoretical)	Very low at sensor nodes due to elimination of idle listening and UAV-scheduled transmissions	Higher UAV-side energy due to continuous cooperation and relay selection overhead	Moderate; optimized at node level but suffers under UAV mobility assumptions
Latency characteristics (Theoretical)	Low and predictable due to cluster scheduling and UAV-controlled synchronization	Variable latency depending on UAV-to-UAV link dynamics	Low under static-sink model but increases significantly if adapted to mobile/UAV sinks
Strengths	Highly energy-efficient in dense WSN; scalable within a single UAV-assisted cell	Strong aerial communication reliability and cooperative diversity	Fine-grained per-node adaptability for deviation-driven networks
Main limitations	Not designed for multi-UAV cooperative routing; cluster-level adaptation	Not applicable to ground WSN data collection scenarios	Not suitable for UAV-assisted networks or mobile sinks

In addition to the mathematical-model/simulation results, a concise theoretical comparison is provided in Table 7 with two recent state-of-the-art MAC protocols-Wu et al.^10^ and Tolani et al.^1^. Since these protocols were designed for fundamentally different environments (multi-UAV FANETs and static-sink WSNs, respectively), they cannot be directly simulated in our single-cell UAV-assisted WSN setting without major redesign. Nevertheless, the comparison highlights that EE-UAV-TLH achieves lower node-side energy consumption through idle-listening elimination and UAV-controlled scheduling, while the referenced protocols would incur higher UAV energy (Wu et al.) or increased latency if adapted to mobility (Tolani et al.). This reinforces that EE-UAV-TLH is specifically optimized for dense, energy-constrained UAV-assisted WSNs, and that theoretical comparison provides a fair and meaningful assessment of strengths and limitations within the intended system model.

## Conclusion

Energy-Efficient UAV-assisted Two-Layer Hierarchical (EE-UAV-TLH) MAC protocol is proposed and analyzed against various frameworks, i.e., TLH-TDMA, TLH-ETDMA, TLH-BMA protocols under different network settings. The simulation results, performed using MATLAB 2024b https://in.mathworks.com/videos/r2024b-release-highlights-1725868702072.html, analyzed the role of critical network parameters,i.e., the number of nodes, event generation probability, and number of network cells over the total energy consumed. It turns out that the TLH-BMA takes advantage of dynamic bit-mapped access mechanism, and can achieve a better energy efficiency performance than its TLH-TDMA and TLH-ETDMA under light traffic and lower node density. In case of a low event generation probability(e. g 0.1), BMA showed the lowest energy consumption, using its selective access scheme to generate empty slots or channels. For high event generation probability (e.g. 0.8), however, BMA’s energy performance deteriorated and it became less energy-efficient than TLH-ETDMA due to its high contention period, particularly when the number of nodes in the network was large indicating the protocol’s traffic load susceptibility. Moreover, by scaling the number of nodes and the cell numbers, TLH-BMA consistently demonstrates a lower energy footprint than the other two protocols, confirming its scalability for large-scale, cluster-based WSNs. By means of UAV-based data collection, the two-layer hierarchical architecture could not only decrease the long distance multi-hop transmission overhead, but also achieve the better load balance of cluster heads. The overall results concludes that the TLH-ETDMA is best suited method for high density nodes and high data traffic and TLH-BMA is best suited method for low data traffic and low density of nodes.

## Data Availability

No datasets were used or generated during the current study.
